# Chemical-Induced Cleft Palate Is Caused and Rescued by Pharmacological Modulation of the Canonical Wnt Signaling Pathway in a Zebrafish Model

**DOI:** 10.3389/fcell.2020.592967

**Published:** 2020-12-14

**Authors:** Rika Narumi, Shujie Liu, Naohiro Ikeda, Osamu Morita, Junichi Tasaki

**Affiliations:** ^1^R&D, Safety Science Research, Kao Corporation, Kawasaki, Japan; ^2^R&D, Safety Science Research, Kao Corporation, Ichikai-machi, Japan

**Keywords:** teratogen, environmental factors, cleft palate, canonical Wnt signaling pathway, zebrafish

## Abstract

Cleft palate is one of the most frequent birth defects worldwide. It causes severe problems regarding eating and speaking and requires long-term treatment. Effective prenatal treatment would contribute to reducing the risk of cleft palate. The canonical Wnt signaling pathway is critically involved in palatogenesis, and genetic or chemical disturbance of this signaling pathway leads to cleft palate. Presently, preventative treatment for cleft palate during prenatal development has limited efficacy, but we expect that zebrafish will provide a useful high-throughput chemical screening model for effective prevention. To achieve this, the zebrafish model should recapitulate cleft palate development and its rescue by chemical modulation of the Wnt pathway. Here, we provide proof of concept for a zebrafish chemical screening model. Zebrafish embryos were treated with 12 chemical reagents known to induce cleft palate in mammals, and all 12 chemicals induced cleft palate characterized by decreased proliferation and increased apoptosis of palatal cells. The cleft phenotype was enhanced by combinatorial treatment with Wnt inhibitor and teratogens. Furthermore, the expression of *tcf7* and *lef1* as a readout of the pathway was decreased. Conversely, cleft palate was prevented by Wnt agonist and the cellular defects were also prevented. In conclusion, we provide evidence that chemical-induced cleft palate is caused by inhibition of the canonical Wnt pathway. Our results indicate that this zebrafish model is promising for chemical screening for prevention of cleft palate as well as modulation of the Wnt pathway as a therapeutic target.

## Introduction

Cleft palate and/or lip is one of the most frequent birth defects, occurring in 1 out of 800 to 2500 live births, and induces severe eating and speaking problems, dental defects, ear infections, and hearing loss (Parker et al., 2010; Mezawa et al., 2019). The patients require long-term treatments, including surgeries, dental treatment, speech rehabilitation and psychological treatment, which impose huge a lifetime burden estimated at $200,000 on their family and social support system (Boulet et al., 2009; Wehby and Cassell, 2010). Thus, prevention and treatment of cleft palate is a worldwide health and medical issue. Cleft palate has complicated etiology and its causation involves both genetic and environmental risk factors (Dixon et al., 2011). To date, over 500 Mendelian syndromes have been reported as congenital diseases, including cleft palate, in Online Mendelian Inheritance in Man (OMIM)^1^, and also over 60 chemicals have been categorized as teratogens that induce cleft palate in ToxRefDB (Martin et al., 2009). Recent studies point out that cleft palate patients have various mutations in components of specific signaling pathways, i.e., the Wnt, Hedgehog, FGF, and TGF-beta signaling pathways (Riley et al., 2007; Lipinski et al., 2010; Liu and Millar, 2010; Parada and Chai, 2012; Stanier and Pauws, 2012; Iwata et al., 2014; Kurosaka, 2015; Reynolds et al., 2019). Thus, for prenatal prevention and therapy of cleft palate, regulation of such signaling pathways will be a central target. In a mouse model with a consistent cleft palate phenotype, the cleft phenotype is partially or completely recovered by chemical modulation of the canonical Wnt signaling pathway (Liu et al., 2007; Jia et al., 2017; Li et al., 2017).

Prenatal exposure to environmental risk factors such as alcohol, cigarette smoking, pharmaceuticals and chemical reagents also leads to cleft palate in mammals, including humans (Shaw et al., 1996; Brent, 2004; DeRoo et al., 2008; Beaty et al., 2016). Some of these chemicals are reported to target the Wnt signaling pathway: excess retinoic acid or dexamethasone exposure induces cleft palate via inhibition of the canonical Wnt pathway in a mouse model (Hu et al., 2013; Okano et al., 2014; Wang et al., 2019). These reports suggest that chemical modulation of the Wnt signaling pathway will be a promising approach for prenatal prevention of cleft palate. The mammalian model has enabled great progress for investigating the etiology and pathology of cleft palate. However, at present, there are few therapies or medications directed at reducing the risk of cleft palate during prenatal development. Thus, a zebrafish screening model could provide another approach for prenatal prevention of cleft palate as well as teratogenicity testing aimed at preventing cleft palate.

As an emerging model organism for human disease, zebrafish provides excellent opportunities for investigating fundamental mechanisms causing common birth defects as well as screening and discovering small molecules that impact human disease (Mork and Crump, 2015; Rennekamp and Peterson, 2015; Wiley et al., 2017; Weinberg et al., 2018; Patton and Tobin, 2019). Zebrafish has several experimental advantages for high-throughput genetic and chemical screening, including evolutionarily conserved developmental programs, cost effectiveness, rapid external development, and transparency during the embryonic stage. Zebrafish ethmoid plate is considered to be a mammalian palate model because the zebrafish palate is composed of cells derived from the frontonasal and maxillary domain, which is similar to the palatogenesis of mammals (Jugessur et al., 2009; Kague et al., 2012; Mongera et al., 2013; Mork and Crump, 2015; Duncan et al., 2017). Moreover, the fundamental signaling pathways and cellular events during craniofacial development, including palatogenesis, are conserved between fish and mammals (Swartz et al., 2011). Recently, the responsible genes, such as *irf6*, *tgf*β*3*, *smad5* and *faf1*, causing cleft palate have been identified, and disruption of these genes is phenocopied in the zebrafish model, validating the genetic relevance of cleft palate in zebrafish to mammalian cleft palate (Cheah et al., 2010; Ghassibe-Sabbagh et al., 2011; Swartz et al., 2011; Dougherty et al., 2013). Furthermore, genetic disruption of the Wnt signaling pathway in zebrafish causes craniofacial anomalies such as cleft palate and micrognathia, suggesting involvement of the Wnt signaling pathway in cleft palate both in fish and mammals (Curtin et al., 2011; Jackson et al., 2015; Duncan et al., 2017; Neiswender et al., 2017). This accumulating knowledge of the genetic phenocopying of cleft palate in the zebrafish palate model indicates the relevance of this model to mammalian cleft palate.

Recently we reported that in a zebrafish model, several teratogens recapitulated craniofacial anomalies found in mammals and some defects phenocopied neurocristopathy, indicating that the zebrafish model is amenable for high-throughput prediction of teratogen-induced craniofacial anomalies of mammals (Liu et al., 2020). Many teratogens that induce cleft palate have been identified by epidemiological studies or teratogenicity testing; however, only a few chemicals, such as retinoic acid and alcohol, have been employed for analyzing detailed cellular and molecular mechanisms of cleft palate in a mammalian model (Kietzman et al., 2014; Okano et al., 2014; Wang et al., 2019). For use in chemical screening for prenatal prediction and prevention of chemical-induced cleft palate, a zebrafish model should be useful in addition to a mouse model, which has been used to detect phenocopied cleft palate and its rescue based on mechanistic insights (Sive, 2011; Hahn and Sadler, 2020). Although our previous study suggested that teratogen-treated zebrafish phenocopied mammalian craniofacial anomalies, the biological relevance of chemical-induced cleft palate in this zebrafish model to mammals still remained unclear. Notably, the causal relationship between the canonical Wnt signaling pathway and chemical-induced cleft palate remained to be examined.

In the present study, we consolidate our previous proof-of-principle study by Liu et al., 2020 showing that zebrafish can be utilized as a discovery platform to investigate chemical-induced neurocristopathies. Zebrafish embryos were exposed to 12 chemical compounds (caffeine, 5-fluorouracil, salicylic acid, hydroxyurea, warfarin, valproic acid, methotrexate, imatinib, thalidomide, phenytoin, dexamethasone, and retinoic acid), which have been identified as teratogens causing cleft palate by epidemiological research and teratogenicity testing in mammals (Table 1). All 12 teratogens induced a series of palate malformations of zebrafish palate dose-dependently, suggesting the phenotypic relevance of this zebrafish model to chemical-induced cleft palate in mammals. Moreover, IWP-L6, a specific inhibitor of the canonical Wnt signaling pathway, also induced clefting phenotypes, which were enhanced by combinatorial treatment with the Wnt inhibitor and teratogens. These results suggested that teratogens inhibited the canonical Wnt signaling pathway, which was confirmed by observation of decreased expression levels of downstream effectors of canonical Wnt signaling (*tcf7* and *lef1*). Cellular defects of teratogen-induced cleft palate were characterized by inhibition of cell proliferation and viability in the palate. Furthermore, cleft palate induced by teratogens was abrogated by three Wnt agonists (BIO, CHIR-99021, and WAY-262611). These findings suggest that inhibition of canonical Wnt signaling in the zebrafish model contributes critically to chemical-induced cleft palate, which is the same etiology as in mammalian models. Taken together, our findings indicate that the zebrafish palate is a suitable model for investigating the etiology of chemical-induced cleft palate as well as for high-throughput chemical screening for prevention of cleft palate and teratogenicity. Additionally, our results provide insights into chemical modulation of the canonical Wnt signaling pathway as a potential target for prenatal prevention of cleft palate.

**TABLE 1:** Chemicals used in the current study.

**Category**	**Compound**	**Abbreviation**	**Birth defects**		**References**
				
			**Rodents**	**Human**	
Antiepileptic drug	Valproic acid	VPA	Cleft palate, spina bifida occulta, and delay in ossification	Cleft palate, spina bifida, atrial septal defect, and hypospadias	Faiella et al., 2000; Jentink et al., 2010
	Phenytoin	PHT	Cleft lip, tetralogy of Fallot, short neck, and diaphragmatic hernia	Cleft lip and palate, congenital heart disease, and microcephaly	Speidel and Meadow, 1972; Czeizel, 1976
Antithrombotic drug	Warfarin	WAF	Maxillonasal hypoplasia and skeletal abnormalities	Cleft palate, nasal hypoplasia, and skeletal abnormalities	Howe and Webster, 1992; Starling et al., 2012; D’Souza et al., 2017
Antineoplastic drug	5-Fluorouracil	5FU	Cleft lip and/or palate, clubbed leg, and polydactyly	Radial aplasia, imperforate anus, esophageal aplasia, and hypoplasia	Stephens et al., 1980; National Toxicology, 2008
	Hydroxyurea	HU	Cleft palate, cleft lip, exencephaly, and clubbed leg	–	Chaube and Murphy, 1966
	Imatinib	IM	Cleft lip, exencephaly, and contraction of forelimb	Cleft palate, polydactyly, hypospadias, scoliosis, and small exomphalos	Pye et al., 2008; El Gendy et al., 2015
	Methotrexate	MTX	Cleft palate, skull defects, and severe fore- and hindlimb dysplasia	Cleft palate, ear malformation, and multiple cardiac malformations	Jordan et al., 1977; Granzow et al., 2003
Immunosuppressive drug	Thalidomide	THA	–	Limb defects, cleft lip and palate, ear defects, and small eyes	Newman, 1985; Speirs, 1962; Taussig, 1962; Smithells and Newman, 1992
Anti-inflammatory drug	Dexamethasone	DEX	Cleft lip, and cleft palate	Cleft lip and palate	Hviid and Mølgaard-Nielsen, 2011; Zhou et al., 2011; Kemp et al., 2016
	Salicylic acid	SA	Cleft palate, spina bifida, cranioschisis, spondyloschisis, and abdominal fissure	Neural tube defects, gastroschisis, and cleft lip/palate	Trasler, 1965; Tagashira et al., 1981; Kozer et al., 2002
Non-pharmaceutical chemical	Caffeine	CAF	Cleft palate, and digital defect	Cleft palate, hydrocephalus, and interventricular septal defect	Nishimura and Nakai, 1960; Collier et al., 2009
	Retinoic acid	RA	Cleft lip and palate, ear defects, and limb and lower-body duplications	Cleft palate, ear defects, hydrocephalus, and teratology of Fallot	Benke, 1984; Lammer et al., 1988; Rutledge et al., 1994; Padmanabhan and Ahmed, 1997
	Boric acid	BA	Rib defects (short rib and wavy rib)	-	Moore, 1997

*All chemicals have been reported to induce birth defects in rodents and human.*

## Materials and Methods

### Zebrafish Maintenance

Zebrafish (*Danio rerio*), strain RIKEN WT (RW), were maintained with a 14-h light/10-h dark cycle and water temperature at 28 (±1)°C. Water quality conditions were maintained according to The Zebrafish Book (Westerfield, 2000) and the Guide for the Care and Use of Laboratory Animals 8th edition (National Research Council, 2011).

### Test Compounds

Test compounds used in this study are listed in Table 1. These tested compounds are known to be teratogens inducing cleft palate in mammals and have been classified into various categories as a result of being tested in zebrafish experiments or chemical safety assays (Hillegass et al., 2008; Selderslaghs et al., 2009; Ito and Handa, 2012; Lee et al., 2012; Teixido et al., 2013; Yamashita et al., 2014; Inoue et al., 2016; Martinez et al., 2018; Cassar et al., 2019). The test compounds and exposure concentrations were determined based on Liu et al., 2020. The exposure concentrations were as follows: hydroxyurea (1 mM, Sigma-Aldrich), valproic acid (7.5–30 μM, Wako), salicylic acid (100–400 μM, Wako), boric acid (1 mM, Wako), and caffeine (0.5–2 mM, Wako), which were diluted from stock solutions prepared with distilled water (Life Technologies), and imatinib (250 μM, Tokyo Chemical Industry), retinoic acid (10–50 nM, Tokyo Chemical Industry), thalidomide (400 μM, Tocris Bioscience), methotrexate (50–200 μM, Wako), warfarin (15–60 μM, Wako), phenytoin (1 mM, Wako), dexamethasone (1 mM, Wako), 5-fluorouracil (1 mM, Wako), and isoniazid (1 mM, LKT Laboratories), which were diluted from stock solutions prepared with dimethyl sulfoxide (DMSO, Wako).

### Egg Production and Chemical Exposure

Adult male and female zebrafish (4–10 months after fertilization) were placed in a breeding tank with a separator in the late afternoon the day before spawning. The separator was removed in the morning and spawning was stimulated when the light was turned on. Fertilized eggs were collected within 1 h after removal of the separator. The eggs were incubated in E3 medium (5 mM NaCl, 0.17 mM KCl, 0.33 mM CaCl_2_, 0.33 mM MgSO_4_, 0.1 mM NaOH) at 28°C and dechorionated by 1 mg/mL Protease type XIV (Sigma-Aldrich) for 10 min at room temperature and washed several times with E3 medium. Dechorionation was done within an hour after fertilization. The dechorionated embryos were incubated with E3 medium and were exposed to the test compounds at 4 hpf. Embryos were treated with Wnt antagonist (IWP-L6, 15 μM, Merck) or Wnt agonist [BIO (100 nM, Sigma-Aldrich), CHIR99021 (300 nM, Abcam) or WAY-262611 (250 nM, Wako)]. Embryos were treated with these small molecules starting at 50 hpf, when the onset of palatogenesis occurs (DeLaurier et al., 2012). The exposure medium was replaced daily and samples were collected at 96 hpf.

### Fluorescence Imaging and Immunofluorescence Staining

Prior to nucleic staining, zebrafish embryos were fixed at 96 hpf with 4% paraformaldehyde (PFA, Wako) for 1 h and Alcian blue cartilage staining was performed as previously described (Liu et al., 2020). Samples were washed twice with PBS-T (phosphate buffered saline containing 0.1% Triton X-100) for 5 min and stained with DAPI (1/5000, DOJINDO) diluted with PBS-T on a shaker for 1 h. After nucleic staining, samples were dissected with fine forceps and embedded in 1% low-melting agarose (Sigma-Aldrich) and then mounted on a 35-mm non-coated glass bottom dish (Matsunami).

For immunofluorescence staining, zebrafish embryos were fixed at 96 hpf with 4% PFA for 1–2 h(s). Samples were washed twice with PBS-T for 5 min and placed in 100% ice-cold methanol (MeOH, KANTO CHEMICAL) and stored for more than 2 h at −20°C to accomplish complete dehydration. Then, samples were gradually rehydrated with 75%, 50%, 25% MeOH in PBS-T (volume percent) for 5 min per wash on ice and processed to remove pigmentation by bleaching in 3% hydrogen peroxide and 0.5% potassium hydroxide under light. After bleaching, samples were incubated in 10 μg/mL Protease type XIV (Sigma-Aldrich) in PBS-T for 30 min and then post-fixed with 4% PFA for 20 min. Samples were washed with 150 mM Tris–HCl (pH 8.5) for 5 min and then heated for 15 min at 70°C following by washing twice with PBS-T for 5 min. Samples were incubated in ice-cold acetone (Wako) for 20 min at −20°C and washed twice with PBS-T for 5 min. Samples were blocked with 3% bovine serum albumin in PBS for 2 h and incubated with rabbit anti-active caspase3 (1/1000, BD Pharmingen: 559565), rabbit anti-phospho-histone H3 (Ser10) (1/500, EMD Millipore: 06-570), mouse anti-collagen type II (anti-coll2, 1/20, DSHB: AB_528165) primary antibody or lectin PNA Alexa Fluor 488 conjugate (1/1000, Thermo) overnight at 4°C, washed six times with PBS-T for 15 min, and then stained with Alexa Fluor 568-goat anti-rabbit, or Alexa Fluor 647-IgG1 kappa-goat anti-mouse secondary antibodies (1/1000, Life Technologies) for 2 h. After washing six times with PBS-T for 15 min, samples were embedded in 1% low-melting agarose and mounted on a 35-mm non-coated glass bottom dish. All immunofluorescence images were acquired by the Zeiss LSM880 or LSM800 system equipped with Zeiss ZEN black or blue software. Z-sections of the images were stacked by Z-projection (projection type: Max intensity) of ImageJ (National Institutes of Health). All procedures were performed at room temperature unless otherwise specified.

### Quantification of Immunofluorescence Image

After nucleic staining, quantification of palate morphology was performed. The phenotypes were categorized based on the criteria shown in Supplementary Figure 1A. Lengths of zebrafish palate and cleft were analyzed using ImageJ. For quantification of the frequency of pH3- or active caspase3-positive cells, the number of proliferative or apoptotic cells in the palate was normalized to 10^–4^ μm^2^. The area of the palate was measured using the ImageJ Measure option. All analysis was performed after the Z-projection. All experiments were performed in triplicate and sample sizes are stated in each figure legend.

### Dissection, RNA Preparation and RT-qPCR Analysis

Zebrafish embryos were stained with 5 μM diaminofluorescein-FM diacetate (DAF-FM DA, GORYO Chemical) in E3 medium at 28°C overnight to visualize cranial cartilage. After the trunk and yolk were removed, the head region of the embryo was treated with 5 mg/ml pancreatin (Wako) for several minutes at room temperature. The fluorescence-positive region of the head region was dissected using Disponano needles (Saito Medical Instruments) under a fluorescence stereomicroscope. Total RNA of each sample was extracted using Trizol (Invitrogen) and an RNeasy Mini Kit (QIAGEN) followed by reverse transcription with a QuantiTect Reverse Transcription Kit (QIAGEN). RT-qPCR analysis was performed with TaqMan Master Mix (Thermo Fisher Scientific), TaqMan probes and gene-specific primers for *tcf7l1a*, *lef1*, and *gapdh* (Bio-Rad) using the 7500 Fast Real-Time PCR System (Thermo Fisher Scientific). For data analysis, relative quantification analysis was performed using the comparative CT (2^–ΔΔCT^) method. For each sample, mRNA levels of the target genes were normalized to the *gapdh* mRNA.

### Statistics

Two-tailed Welch’s *t*-tests were used to determine *P-*values for RT-PCR experiments. Multiple comparison tests were performed using Graph Pad Prism version 8 for Windows (La Jolla). *P*-values were calculated with one-way ANOVA followed by Dunnett’s multiple comparison tests for quantification of pH3- and Cas3-positive cells. *P-*values less than 0.05 were considered to be statistically significant. All data are presented as the mean ± SD unless otherwise specified.

## Results

### Chemical-Induced Cleft Palate Is Recapitulated in Zebrafish Embryo Model

**FIGURE 1:**
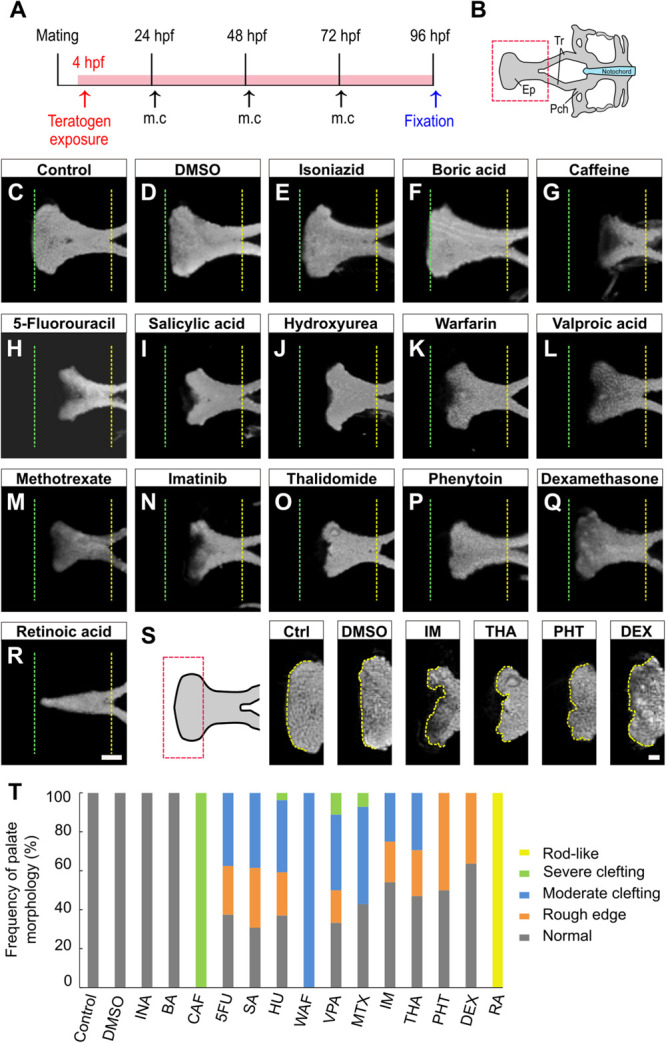
Morphological phenotype of chemical-induced cleft palate in zebrafish embryos. **(A)** Experimental time course m.c.: medium change. **(B)** Atlas of the neurocranial cartilage. Ep, Ethmoid plate; Tr, Trabecula; Pch, Parachordal. **(C–R)** Fluorescence images of the ethmoid palate (zebrafish palate) at 96 hpf. Nuclei of cartilage cells were stained with DAPI. The anterior and posterior edges of the palate are indicated by green and yellow dotted lines, respectively. Exposure concentration was as follows: DMSO as vehicle control (0.1%), isoniazid (INA, 1 mM), boric acid (BA, 1 mM), caffeine (CAF, 1 mM), 5-fluorouracil (5FU, 1 mM), salicylic acid, (SA, 200 μM), hydroxyurea (HU, 1 mM), warfarin (WAF, 30 μM), valproic acid (VPA, 15 μM), methotrexate (MTX, 200 μM), imatinib (IM, 250 μM), thalidomide (THA, 400 μM), phenytoin (PHT, 1 mM), dexamethasone (DEX, 1 mM), and retinoic acid (RA, 10 nM). **(S)** Highly magnified images of the anterior edge of the palate. Ctrl, control; IM, imatinib; THA, thalidomide; PHT, phenytoin; DEX, dexamethasone. Yellow dotted line traces the shape of anterior edge. **(T)** Frequency of palate morphology. *n* = 19 (Control), 18 (DMSO), 11 (INA), 14 (BA), 17 (CAF), 8 (5FU), 16 (SA), 27 (HU), 11 (WAF), 18 (VPA), 14 (MTX), 24 (IM), 17 (THA), 10 (PHT), 22 (DEX), 16 (RA). Scale bars: 50 μm in **(C–R)**, 20 μm in **(S)**.

To examine the effects of teratogens on zebrafish palatogenesis, embryos from blastula to larval stage (4–96 hpf) were exposed to 14 chemical reagents, which included 12 teratogens that induce cleft palate, a teratogen that does not induce cleft palate and a non-teratogen, according to (Kimmel et al., 1995; Figure 1A and Table 1). These teratogens have been reported to induce orofacial clefts in humans and/or rodents. Morphological analysis of the palate was carried out at 96 hpf by nuclear staining after soft tissue removal and dissection of the neurocranium (Figures 1A,B). All teratogens induced palate malformation, which was classified into four types of defects as follows: rough edge, moderate clefting, severe clefting, and rod-like (Supplementary Figure 1A). Cleft at the center of the anterior edge of the palate (moderate clefting and severe clefting) was frequently observed in CAF-, 5FU-, SA-, HU-, WAF-, VPA-, and MTX-treated embryos (Figures 1C,D,G–M). Rough edge consisting of several small clefts was frequently induced by IM, THA, PHT, and DEX treatment (Figures 1C,D,N–Q,S). These palatal defects were quantified and summarized in Figure 1T. Dose-response analysis revealed that these phenotypes were observed in a dose-dependent manner (Supplementary Figure 1B). RA induced a rod-like malformation (Figure 1R). This defect was only observed in RA-treated embryos (Figure 1T and Supplementary Figure 1B). Non-teratogens (DMSO as vehicle control and isoniazid; INA) and a teratogen that does not induce cleft palate (boric acid; BA) did not induce palate abnormalities (Figures 1C–F,T). These results suggest that specific teratogens induce cleft palate and chemical-induced cleft palate is recapitulated in the zebrafish model.

### Inhibition of the Canonical Wnt Signaling Pathway Induces Chemical-Induced Cleft Palate

**FIGURE 2:**
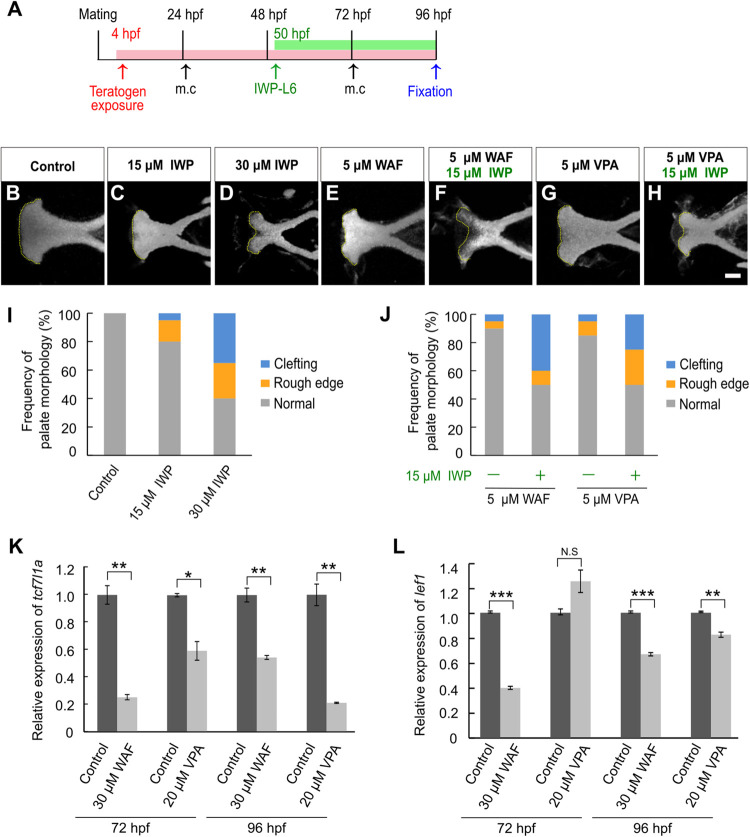
Chemical-induced cleft palate was induced by inhibition of the canonical Wnt signaling pathway. **(A)** Experimental time-course. m.c.: medium change. **(B–H)** Fluorescence images of the palate at 96 hpf. Nuclei of cartilage cells were stained with DAPI. Yellow dotted line indicates the anterior edge of the palate. **(I,J)** Frequency of palate morphology. *n* = 20 for each sample. **(K,L)** Quantification of relative levels of *tcf7l1a* **(K)** and *lef1* **(L)** mRNA isolated from the neurocranium at 72 and 96 hpf. Each mRNA level was normalized by *gapdh* mRNA by the comparative CT (2^– ΔΔ*CT*^) method. Data are shown as mean ± SD from triplicate experiments. *P < 0.05, **P < 0.01, ***P < 0.001 (two-tailed Welch’s *t*-tests). Scale bar: 50 μm.

Next, we investigated the contribution of canonical Wnt signaling to chemical-induced cleft palate because the canonical Wnt signaling pathway is critically associated with cleft palate in mammals (Song et al., 2009; Kurosaka et al., 2014; Reynolds et al., 2019). We performed morphological analysis after pharmacological inhibition of the canonical Wnt signaling pathway using a specific Porcn inhibitor, IWP-L6 (Wang et al., 2013; Grainger et al., 2016). To focus on the effect on the zebrafish palate, IWP-L6 added specifically during palatogenesis (50–96 hpf) (Figure 2A). Cleft phenotypes were observed in IWP-L6-treated embryos dose-dependently (Figures 2B–D,I). Moreover, these palatal malformations were phenocopied by teratogen-treated embryos (Figures 1G–Q), suggesting that the teratogens disturbed the canonical Wnt signaling pathway. To test this, combinatorial treatment with the Wnt antagonist and the teratogens was performed (Figure 2A). Warfarin (WAF) and valproic acid (VPA) were selected as suitable chemicals among the teratogens to verify the combinatorial effect on cleft palate because of their dose-dependent phenotypic severity and their different pharmacodynamics (Supplementary Figure 1B; Holford, 1986; Ghodke-Puranik et al., 2013). The low dosage of WAF (5 μM) and VAP (5 μM) induced a small number of palatal defects (Figures 2E,G,J). Combinatorial treatment with a low dose of IWP-L6 (15 μM, which alone induced a small number of palatal defects) increased the number of palatal defects in the treated embryos (Figures 2F,H,J). Furthermore, to obtain a readout of the effect of disturbing the canonical Wnt signaling pathway, we analyzed the expression levels of *transcription factor 7* (*tcf7*) and *lymphoid enhancer binding factor 1* (*lef1*), which are two of the downstream effectors by quantitative real-time PCR (Veien et al., 2005; Wang et al., 2009; Hagemann et al., 2014). After WAF and VAP treatment, the neurocranium was dissected to enrich for the neurocranial progenitors at 72 and 96 hpf, at which time basic morphogenesis of the palate is completed (DeLaurier et al., 2012). The expression levels of both *tcf7l1a* and *lef1* in the neurocranium were significantly reduced by teratogen exposure (Figures 2K,L). These results suggest that inhibition of canonical Wnt signaling contributes to chemical-induced cleft palate in zebrafish.

### Decreased Proliferation and Increased Apoptosis Are Observed in Chemical-Induced Cleft Palate

**FIGURE 3:**
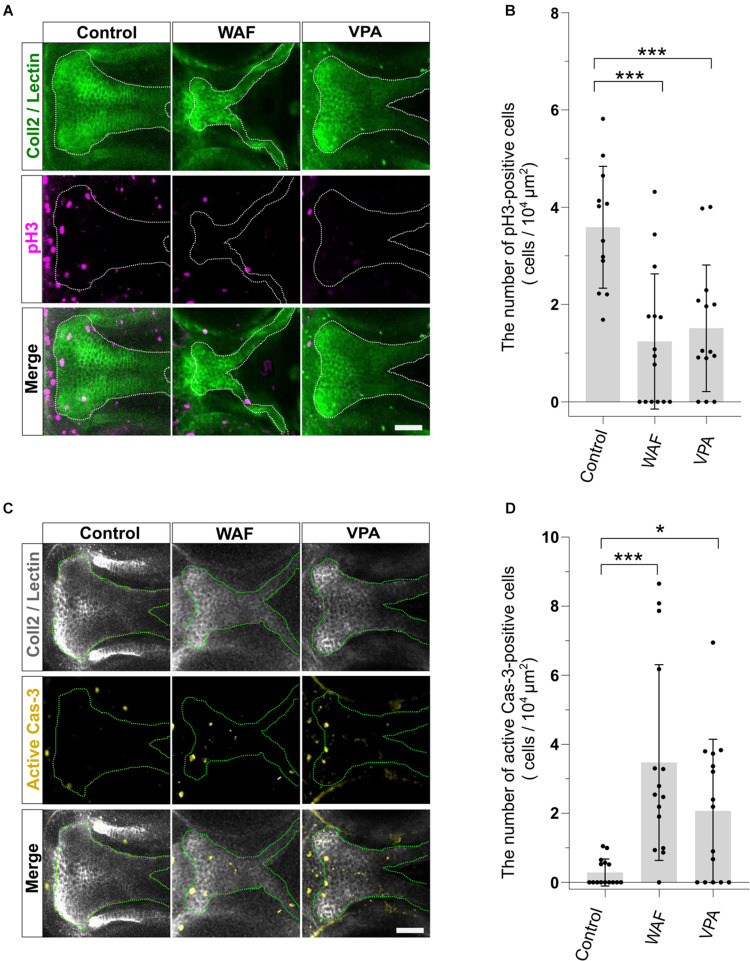
The pattern of proliferation and apoptosis in the cleft palate induced by teratogens. **(A)** Immunofluorescence images of proliferative cells in the palate at 96 hpf. Embryos were treated with WAF (30 μM) or VPA (20 μM) and stained with anti-coll2 antibody, lectin PNA and anti-phospho-histone H3 (pH3) antibody. Green indicates cartilage cells double stained with anti-coll2 antibody and lectin PNA. White dotted lines trace the shape of the palate. Magenta indicates proliferative cells stained with anti-pH3 antibody. **(B)** The number of pH3-positive cells in the palate. Numerical value is normalized by 10^4^ μm^2^. *n* = 12 (Control), 15 (WAF), 14 (VPA), ***P < 0.001 (one-way ANOVA followed by Dunnett’s multiple comparison test). **(C)** Immunofluorescence images of apoptotic cells in the palate at 96 hpf. Embryos were treated with WAF (30 μM) or VPA (20 μM) and stained with anti-coll2, lectin PNA anti-active caspase 3 (active Cas-3) antibody. White indicates cartilage cells double stained with anti-coll2 antibody and lectin PNA. Green dotted lines trace the shape of ethmoid plate. Yellow indicates apoptotic cells stained with anti-caspase3 antibody. **(D)** The number of active caspase3-positive cells in the palate. Numerical value was normalized to 10^4^ μm^2^. *n* = 15 (Control), 15 (WAF), 15 (VPA), *P < 0.05, ***P < 0.001 (one-way ANOVA followed by Dunnett’s multiple comparison test). Scale bars: 50 μm.

The proper regulation of cell proliferation and cell death is one of the key factors for developing the proper size and shape of organs during morphogenesis, and disruption of such regulation of proliferation and apoptosis in palatal shelves induces cleft palate in mammalian models (He et al., 2011; Bush and Jiang, 2012). Thus, the induction of cleft palate by teratogens raised the possibility that cell proliferation and/or viability were inhibited by the teratogens. To examine the effect of teratogens on cell proliferation and apoptosis, we performed immunofluorescence staining with anti-phospho-Histone H3 (pH3) antibody as a mitotic marker and anti-active Caspase-3 (active Cas-3) antibody as an apoptosis marker. After WAF and VPA treatment, the numbers of proliferative and apoptotic cells in zebrafish palate were quantified (Figure 3). Palatal morphology was marked by double staining with anti-Collagen type 2 antibody and lectin PNA (Figure 3A). WAF (30 μM) and VAP (20 μM) treatment induced cleft palate and significantly decreased the number of pH3-positive cells in the palate (Figures 3A,B). In contrast, the number of active Cas-3-positive cells was markedly increased in the palate (Figures 3C,D). No cellular changes were observed in the embryos exposed to INA, which is a non-teratogenic chemical (Supplementary Figure 2). These results indicate that disruption of the balance between cell proliferation and apoptosis occurs in teratogen-treated embryos, leading to cleft palate.

### Wnt Agonists Rescue Chemical-Induced Cleft Palate

**FIGURE 4:**
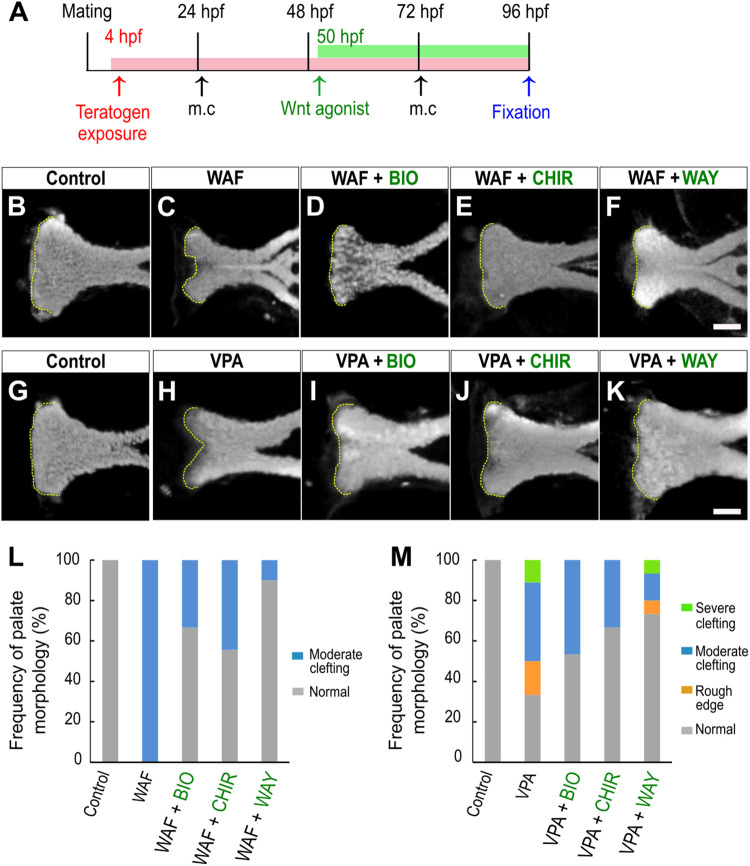
Restoration of chemical-induced cleft palate by Wnt agonists. **(A)** Experimental time course. m.c.: medium change. **(B–K)** Fluorescence images of the palate at 96 hpf. **(B,C)** WAF (30 μM) exposure induced cleft palate. **(D–F)** The cleft palate was rescued by combinatorial treatment with BIO (100 nM), CHIR99021 (300 nM) or WAY-262611 (250 nM). **(G,H)** VPA (20 μM) exposure induced cleft palate. **(I–K)** The cleft palate caused by VPA was rescued by combinatorial treatment with BIO (100 nM), CHIR99021 (300 nM) or WAY-262611 (250 nM). White indicates nuclei stained with DAPI. Yellow dotted lines trace the shape of the anterior edge of the plate. **(L,M)** Frequency of rescued cleft palate. *n* = 18 (Control), 11 (WAF), 12 (WAF + BIO), 9 (WAF + CHIR), 10 (WAF + WAY) in **(L)**, *n* = 19 (Control), 18 (VPA), 15 (VPA + BIO), 18 (VPA + CHIR),15 (VPA + WAY) in **(M)**. Scale bar: 50 μm.

We showed that inhibition of the canonical Wnt signaling pathway is a contributor to chemical-induced cleft palate in our zebrafish model. Several lines of evidence suggested that modulation of the canonical Wnt signaling pathway would induce or correct chemical-induced cleft palate. Next, to test this hypothesis, we analyzed the effect of simultaneous treatment with the teratogens and small molecule Wnt agonists: two glycogen synthase kinase-3 (Gsk-3) inhibitors: (2′Z,3′E)-6-bromoindirubin-3′-oxime (BIO) and CHIR-99021 (CHIR), and one Dickkopf1 (Dkk1) inhibitor: WAY-262611 (WAY) (Figure 4A). These two types of small molecules specifically inhibit components of the canonical Wnt signaling pathway and lead to activation of the Wnt signaling pathway in zebrafish and mammals (Chen et al., 2014; Nishiya et al., 2014; Jia et al., 2017). These agonists were administered from 50 hpf, a critical developmental window for zebrafish palatogenesis (DeLaurier et al., 2012). Cleft phenotype was induced by WAF and VPA exposure (Figures 4B,C,G,H,L,M). These cleft palate phenotypes were rescued by the Wnt agonist treatment (Figures 4D–F,I–K). Moreover, quantitative analysis showed that the frequency of the severe phenotype was reduced by the Wnt agonist treatment (Figures 4L,M). In contrast, the rod-like phenotype induced by RA treatment was not rescued in the presence of the Wnt agonists (Supplementary Figure 3). Therefore, the cleft palate phenotype alone was specifically restored by the Wnt agonist treatment. These results suggest that activation of the canonical Wnt signaling pathway corrects a certain type of cleft palate induced by teratogens.

### Palate Rescued by Wnt Agonists Shows Restored Cell Proliferation and Apoptosis

**FIGURE 5:**
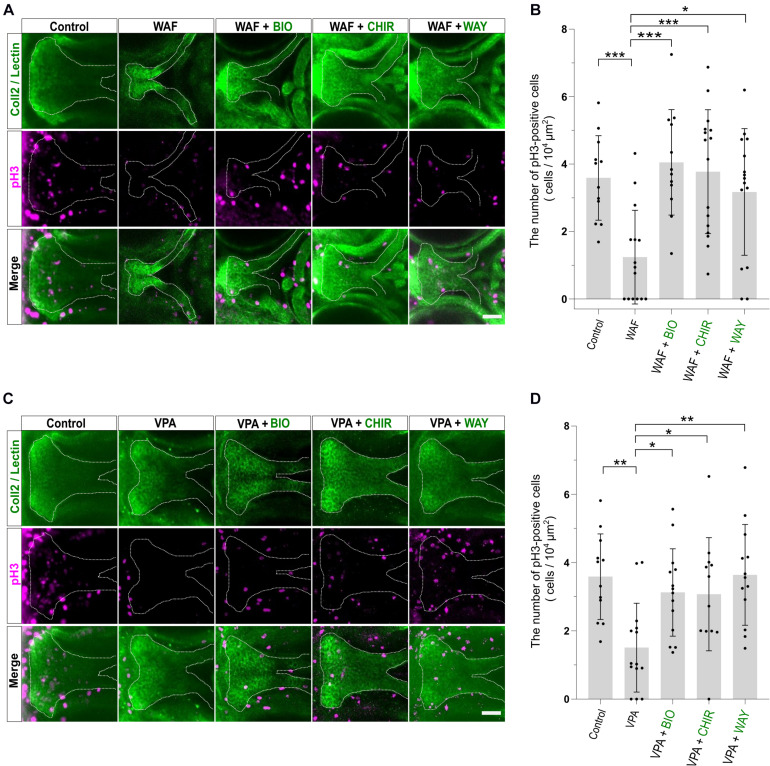
Cell proliferation in the palate was restored by combinatorial treatment with the teratogen and Wnt agonists. **(A)** Immunofluorescence images of proliferative cells of the plate at 96 hpf. Experimental time course is the same as in Figure 4A. WAF (30 μM) exposure induced cleft palate and decreased the number of pH3-positive cells in the palate. The number of pH3-positive cells was restored by combinatorial treatment with BIO (100 nM), CHIR99021 (300 nM) or WAY-262611 (250 nM). Green indicates cartilage cells double stained with anti-coll2 antibody and lectin PNA. White dotted lines trace the shape of the palate. Magenta indicates proliferative cells stained with anti-pH3 antibody. **(B)** Quantification of the number of pH3-positive cells in the palate. Numerical value was normalized to 10^4^ μm^2^. **(C)** VPA (20 μM) exposure induced cleft palate and the number of pH3-positive cells was decreased. The number of pH3-positive cells was restored by combinatorial treatment with BIO (100 nM), CHIR99021 (300 nM) or WAY-262611 (250 nM). **(D)** Quantification of the number of pH3-positive cells in the palate. Numerical value was normalized to 10^4^ μm^2^. *n* = 12 (Control), 15 (WAF), 12 (WAF + BIO), 16 (WAF + CHIR), 15 (WAF + WAY) in **(B)**, *n* = 12 (Control), 14 (VPA), 14 (VPA + BIO), 12 (VPA + CHIR), 13 (VPA + WAY) in **(D)**, *P < 0.05, **P < 0.01, ***P < 0.001 (one-way ANOVA followed by Dunnett’s multiple comparison test). Scale bar: 50 μm.

**FIGURE 6:**
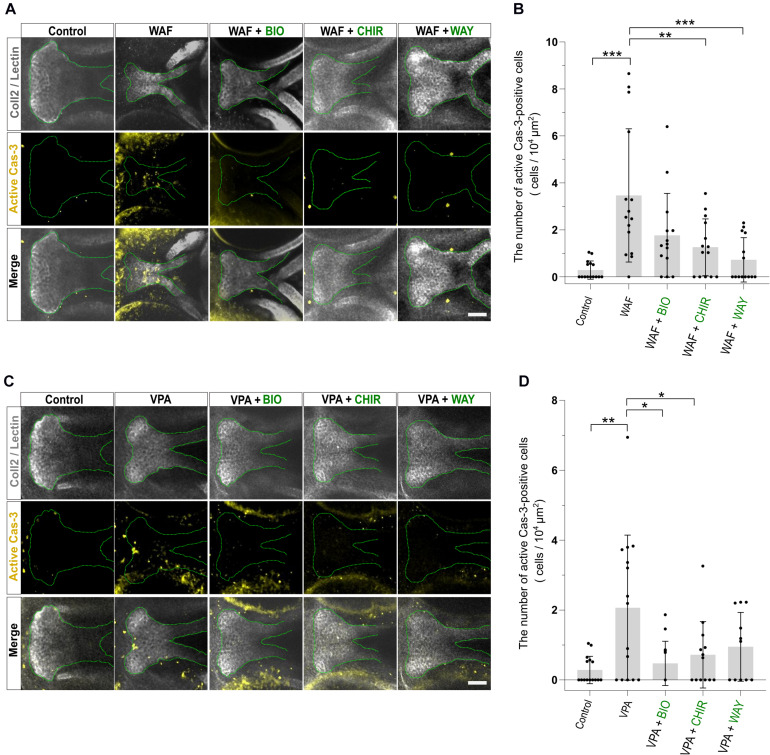
Apoptosis level was restored by combinatorial treatment with teratogen and Wnt agonists. **(A)** Immunofluorescence images of apoptotic cells in the palate at 96 hpf. Experimental time course is the same as in Figure 4A. WAF (30 μM) exposure induced cleft palate and increased the number of active Cas3-positive cells in the palate. The number of active Cas3-positive cells was restored to normal by combinatorial treatment with BIO (100 nM), CHIR99021 (300 nM) or WAY-262611 (250 nM). White indicates cartilage cells double stained with anti-coll2 antibody and lectin PNA. Green dotted lines trace the shape of the palate. Yellow indicates apoptotic cells stained with anti-caspase3 antibody. **(B)** Quantification of the number of active Cas3-positive cells in the palate. Numerical value is normalized to 10^4^ μm^2^. **(C)** VPA (20 μM) exposure induced cleft palate and increased the number of active Cas3-positive cells. The number of active Cas3-positive cells was restored to normal by combinatorial treatment with BIO (100 nM), CHIR99021 (300 nM) or WAY-262611 (250 nM). **(D)** Quantification of the number of active Cas3-positive cells in the palate. Numerical value is normalized to 10^4^ μm^2^. *n* = 15 (Control), 15 (WAF), 14 (WAF + BIO), 14 (WAF + CHIR), 14 (WAF + WAY) in **(B)**, *n* = 15 (Control), 15 (VPA), 14 (VPA + BIO), 13 (VPA + CHIR), 11 (VPA + WAY) in **(D)**, *P < 0.05, **P < 0.01, ***P < 0.001 (one-way ANOVA followed by Dunnett’s multiple comparison test). Scale bar, 50 μm.

Chemical-induced cleft palate was rescued phenotypically by Wnt agonist treatment (Figure 4). Our results showed that teratogens caused decreased cell proliferation and increased apoptosis in the palate and led to disturbance of proper palatogenesis (Figure 3). To confirm the restoration of cell proliferation and apoptosis to the normal levels in the rescued palate, we performed simultaneous treatment with the teratogens and the Wnt agonists, followed by immunostaining with anti-pH3 antibody and anti-active Cas3 antibody (Figures 5, 6). WAF treatment significantly lowered cell proliferation in the palate; however, cell proliferation was restored to a level which appeared adequate for developing normal palatal morphology by BIO, CHIR, and WAY treatment (Figures 5A,B). Restoration of cell proliferation was also observed upon combinatorial treatment with VPA and the Wnt agonists (Figure 5C). Consistent with this, the number of anti-pH3-positive cells recovered as compared with the control (Figure 5D).

Next, we investigated the apoptosis in the recovered palate by anti-active Caspase-3 staining and its quantification. WAF treatment resulted in a significant increase of apoptosis in the zebrafish palate (Figures 6A,B). CHIR and WAY treatment significantly rescued this WAF-induced apoptosis and BIO treatment tended to decrease it (Figures 6A,B). In VPA-exposed embryos, the apoptosis in the palate was significantly increased, and this increase was blocked by BIO, CHIR, and WAY treatment (Figures 6C,D). These results suggest that inhibition of the canonical Wnt signaling pathway contributes to both decreased cell proliferation and increased apoptosis and leads to cleft palate.

## Discussion

**FIGURE 7:**
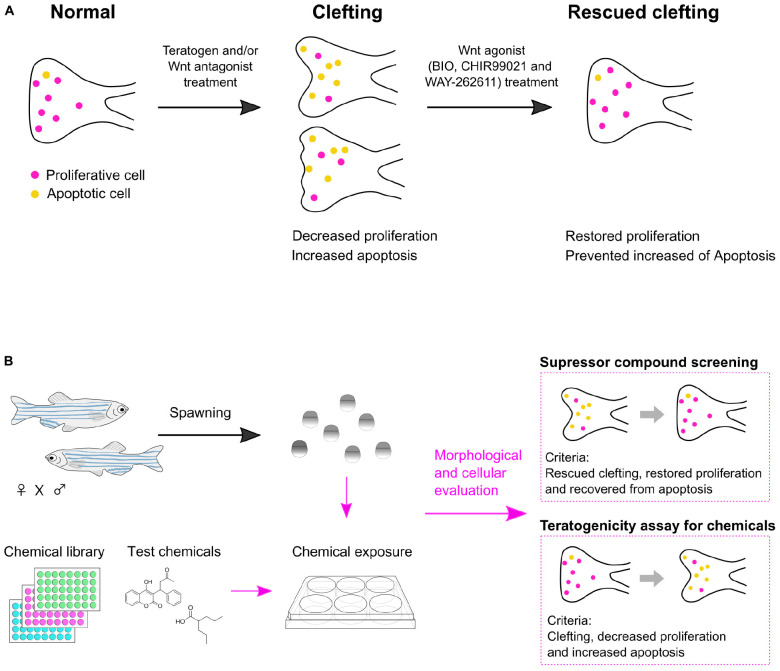
Summary of chemical-induced cleft palate. **(A)** Teratogen induces cleft palate by inhibition of the canonical Wnt signaling pathway. This defect is characterized by decreased proliferation and increased apoptosis in the zebrafish palate. Chemical-induced cleft palate is prevented by Wnt agonist treatment. **(B)** Application of a zebrafish model for suppressor screening as well as teratogenicity assay for chemicals.

Our results revealed that: (1) Cleft palate in zebrafish was specifically induced by the teratogens. (2) Inhibition of the canonical Wnt pathway caused cleft palate. (3) Chemical-induced cleft palate is characterized by decreased cell proliferation and increased apoptosis in the palate. (4) Wnt agonist treatment rescued chemical-induced cleft palate (Figure 7A).

We selected 12 teratogens to examine the relevance of cleft palate in the zebrafish model to mammalian cleft palate. These teratogens are known to induce cleft palate in mammals, including human (Table 1). Zebrafish embryos were exposed to these 12 teratogens, and all of them induced palatal defects dose dependently, ranging from a rough edge to a cleft at the anterior edge of the palate. Furthermore, the zebrafish model showed potential for distinguishing teratogens from non-teratogens. Isoniazid (INA), which is an anti-tubercular drug, does not induce teratogenicity (Snider et al., 1980). Boric acid (BA) is known to be a teratogen, but does not induce cleft palate in mammalian models (Heindel et al., 1994; Price et al., 1996). Zebrafish embryos did not show cleft palate after INA or BA treatment, and precisely detected teratogens inducing cleft palate. BA-treated zebrafish showed other types of teratogenicity, such as micrognathia, reported in mammalian models (Liu et al., 2020). Therefore, although further accumulation of evidence about various chemicals is required to substantiate the validity of the zebrafish model for prediction of chemical-induced cleft palate, our results suggest that the zebrafish model detects phenotypically similar teratogenic responses to mammals’ and indicates conserved responses to teratogens between fish and mammals.

Retinoic acid (RA) alone induced a rod-like phenotype in a dose-dependent manner and did not induce clefting in the palate. It is reported that RA induces holoprosencephaly (HPE), which is a birth defect with various degrees of both defects in the brain and facial abnormalities such as cleft palate, in zebrafish, mouse, and human (Gongal and Waskiewicz, 2008; Maurus and Harris, 2009; Roessler and Muenke, 2010; Billington et al., 2015). It is reported that the palatal morphology of *sonic you* (*syu*) mutant zebrafish embryos, which have disruption of the *sonic hedgehog* gene, and of embryos treated with the Hedgehog (Hh) signaling inhibitor cyclopamine (CyA) show a rod-like structure like that observed in the RA-exposed embryos (Wada et al., 2005). Corresponding to those reports, our previous study showed a decreased expression level of *zic2a*, which is a gene responsible for HPE, in RA-treated embryos (Gongal and Waskiewicz, 2008; Maurus and Harris, 2009; Solomon et al., 2010; Liu et al., 2020). Thus, our results indicate that the zebrafish model detects and recapitulates chemical-induced HPE.

Furthermore, we showed that chemical modulation of the canonical Wnt pathway did not rescue the RA-induced defect, suggesting that RA-induced palatal abnormality was induced as a consequence of complicated signaling cross-talk. The RA signaling pathway plays an essential role in normal palatogenesis and interacts with other signaling pathways, such as the canonical Wnt signaling pathway, Hedgehog signaling pathway and Fgf signaling pathways (Rhinn and Dolle, 2012). In a mouse model, interplay between the Hh signaling pathway and the Wnt signaling pathway is required for cleft palate (Kurosaka et al., 2014). In accord with that previous report, we also found that RA treatment disturbed the expression of downstream effectors of both the canonical Wnt pathway (*tcf7l1a* and *lef1*) and the Hh pathway (*gli1*) (data not shown). This evidence indicates that RA interferes with the Hh pathway and canonical Wnt pathway, which has also been reported in mouse palatogenesis (Hu et al., 2013; Wang et al., 2019). Our finding suggests that partial rescue of the RA phenotype by Wnt agonist treatment could be strengthened to complete rescue by simultaneous activation of the Hh and the Wnt pathway. This evidence indicates that the zebrafish model has potential for analyzing complicated etiology, such as signaling cross-talk, leading to birth defects. Collectively, our findings demonstrate that the zebrafish model detects chemical-induced cleft palate found in mammals and will be applicable mechanism-based prediction of chemical-induced cleft palate.

In addition to the phenotypic relevance, our findings showed that chemical-induced cleft palate is induced by inhibition of the canonical Wnt pathway in the zebrafish model. Low doses of Wnt antagonists enhanced the cleft phenotype induced by teratogens (warfarin: WAF and valproic acid: VPA) and caused decreased expression of downstream effectors of the canonical Wnt signaling pathway. These results imply that teratogens disrupt canonical Wnt signaling, which is a target signaling pathway of cleft palate in mammals (Reynolds et al., 2019). In addition, these results support a previous *in vitro* study that showed that disruption of Wnt signaling is one of the important mechanisms underlying VPA-induced teratogenicity (Langlands et al., 2018). Thus, considering the dose-dependent severity of the chemical-induced cleft phenotypes, our findings indicate that the teratogens examined here target the canonical Wnt pathway. Moreover, we investigated the cellular response associated with Wnt inhibition during palatogenesis. In zebrafish embryos, WAF and VPA exposure inhibited both cell proliferation and viability in the palate. These results suggest that inhibition of the canonical Wnt pathway causes cleft palate via altered cell proliferation and apoptosis in the zebrafish model. Similar observations are also found in mammalian models: Pax9-deficient mice show inhibition of the canonical Wnt pathway as well as retardation of palatal growth marked by decreased cell proliferation (Jia et al., 2017; Li et al., 2017). Another study showed that Wnt-mediated Tgf-β3 activation regulates palatal shelf closure, and inhibition of the Tgf-β3 pathway causes cleft palate via reduced cell proliferation and increased apoptosis (He et al., 2011). Thus, the zebrafish model recapitulates mammalian cleft palate etiology at the cell signaling and cellular levels.

To achieve accurate prediction and reveal the etiology and pathology of chemical-induced cleft palate, detailed analysis of the developmental origin of proliferative and apoptotic cells in chemical-induced cleft palate and target cells adversely affected by teratogens will be needed. The cells composing the zebrafish palate are progenies of neural crest cells migrating from the frontonasal and maxillary domain to form the palate, a process which is conserved between fish and mammals (Swartz et al., 2011; Dougherty et al., 2013; Mork and Crump, 2015). In our previous report, we demonstrated that 12 teratogens disrupted cranial neural crest cell development. Besides chemical-induced craniofacial anomalies, developmental defects in eye, otic vesicles, the heart and/or body axis were observed as a result of teratogen treatment (Liu et al., 2020). Thus, to discriminate teratogen effects on neural crest cells from effects on other cell types will be required in order to eliminate the possibility that secondary effect(s) on neural crest cells development are caused by the teratogens. For this purpose, we have set up experimental systems such as transgenic lines of cranial neural crest cells and target organs for applying spatio-temporal analysis. In addition to utilizing these lines, we will perform teratogen treatment at different time points to define the specific effects on neural crest cells and their descendants. These analyses will reveal whether chemical-induced cleft palate is caused by primary defect(s) in neural crest cells and will provide insights into the relevance to mammals, including human.

We investigated the effect of cleft palate prevention by chemical modulation of the canonical Wnt pathway. Treatment with small molecule Wnt agonists (BIO, CHIR99021, WAY-262611) rescued chemical-induced cleft palate at the morphological and cellular level. This result supports the conclusion that inhibition of Wnt signaling has a significant role in chemical-induced cleft palate via decreased cell proliferation and viability in the zebrafish palate. In a mouse model with cleft palate, Wnt agonist treatment also corrected cleft palate (Liu et al., 2007; Jia et al., 2017; Li et al., 2017). Therefore, our results demonstrate the relevance of our zebrafish model to the development of mammalian cleft palate. However, chemical-induced cleft palate was not completely prevented by chemical modulation of the canonical Wnt pathway, implying insufficient penetrance of the small molecules or the existence of another signaling pathway(s) for complete prevention of cleft palate. To strengthen the prevention of cleft palate, genetic modification of the canonical Wnt signaling could be effective. In addition, modulation of an additional signaling pathway should also be considered. In the zebrafish model, disruption of the non-canonical Wnt pathway also caused cleft phenotype, and cross-talk between the non-canonical and canonical Wnt pathway was previously reported (Curtin et al., 2011; Duncan et al., 2017). To obtain higher efficacy, simultaneous chemical modulation or chemical and genetic modulation with other signaling pathways will be a next step toward prevention of cleft palate. The present results demonstrate the usefulness of the zebrafish model for mechanism-based suppressor chemical screening as well as the promising potential of the Wnt pathway as a therapeutic target.

In this report, we investigated the biological relevance of a zebrafish experimental model of chemical-induced cleft palate to cleft palate in mammals at the morphological, cellular and signaling levels. The results support the usefulness of the zebrafish model for teratogenicity screening. Also, the rescue experiments further indicated the usefulness of the zebrafish model: chemical modulation of the Wnt pathway showed the possibility of using this zebrafish model for chemical screening for prevention or treatment of cleft palate (Figure 7B).

In sum, we show a proof of concept for this zebrafish palate model for chemical screening for prediction and prevention of chemical-induced cleft palate. Also, our findings show that the canonical Wnt signaling pathway would be a therapeutic target.

## Data Availability Statement

The raw data supporting the conclusions of this article will be made available by the authors, without undue reservation.

## Ethics Statement

Ethical review and approval was not required for the animal study because according to the Council Directive 2010/63/EU, zebrafish embryos and larvae up to 5 day old are excepted. However, we performed any experiments and fish husbandry according to the Zebrafish Book (Westerfield, 2000) and the Guide for the Care and Use of Laboratory Animals 8th edition (National Research Council, 2011). Written informed consent was obtained from the owners for the participation of their animals in this study.

## Author Contributions

RN performed conceptualization, methodology, validation, formal analysis, investigation, writing – original draft preparation, writing, and visualization. SL performed methodology and investigation. NI and OM performed original draft preparation. JT performed conceptualization, methodology, validation, formal analysis, writing – original draft preparation, writing – review and editing, visualization, and supervision. All authors contributed to the article and approved the submitted version.

## Conflict of Interest

All authors are employed by the company Kao Corporation.

## Footnotes

1 https://omim.org

## Supplementary Material

Supplementary Figure 1 Phenotypic severity of teratogen treatment in a dose-dependent manner. **(A)** Definition of phenotypic severity. Anterior is to the left. Green dotted lines indicate the anterior **(left)** and posterior **(right)** edge of the zebrafish palate, respectively. The palate length between the green dotted lines is defined as 100% length. Red dotted line indicates 40% length line of the palate length. Each phenotype was defined as follows: rough edge; several small nicks existing at the anterior edge, moderate clefting; under 40% clefting at the center of the palate, severe clefting; over 40% clefting of at the center of the palate, and rod-like; the palate showing rod-like structure. **(B)** Frequency of palatal defects in dose-dependent manner. *n* = 11 (VPA 7.5 μM), 18 (VPA 15 μM), 20 (VPA 30 μM), 10 (WAF 15 μM), 14 (WAF 30 μM), 14 (WAF 60 μM), 10 (SA 100 μM), 13 (SA 200 μM), 13 (SA 400 μM), 11 (MTX 50 μM), 14 (MTX 100 μM), 8 (MTX 200 μM), 13 (CAF 500 μM), 17 (CAF 1 mM), 10 (CAF 2 mM), 16 (RA 10 nM), 12 (RA 30 nM), 11 (RA 50 nM).

Supplementary Figure 2 Proliferation and apoptosis in the palate of INA-exposed embryos. **(A)** Immunofluorescence images of proliferative cells in the palate. Control is the same as Figure 3A. Embryos were treated with isoniazid (1 mM) and then examined by fluorescent immunohistochemistry. No striking difference was observed between the control and treated embryos. Green indicates cartilage cells double stained with anti-coll2 antibody and lectin PNA. White dotted lines trace the shape of the palate. Magenta indicates proliferative cells stained with anti-pH3 antibody. **(B)** Immunofluorescence images of apoptotic cells in the palate at 96 hpf. Control is the same as in Figure 3C. Embryos were treated with isoniazid (1 mM) and then examined by fluorescent immunohistochemistry. No striking difference was observed between the control and treated embryos. White indicates cartilage cells double stained with anti-coll2 antibody and lectin PNA. Green dotted lines trace the shape of the palate. Yellow indicates proliferative cells stained with anti-caspase3 antibody. Scale bar: 50 μm.

Supplementary Figure 3 Rod-like phenotype was not rescued by Wnt agonists. **(A–E)** Fluorescence images of palate at 96 hpf. Nuclei of cartilage cells were stained with DAPI. Embryos were treated with retinoic acid (RA, 10 nM). **(A,B)** Retinoic acid (RA) induced rod-like phenotype. **(C–E)** The rod-like phenotype was not rescued by combinatorial treatment with Wnt agonists [BIO (100 nM), CHIR99021 (300 nM) or WAY-262611 (250 nM)]. Scale bar: 50 μm.
