# Supplementary figures and images for: Chemical-Induced Cleft Palate Is Caused and Rescued by Pharmacological Modulation of the Canonical Wnt Signaling Pathway in a Zebrafish Model
Source: Front Cell Dev Biol. 2020 Dec 14;8:592967. doi: 10.3389/fcell.2020.592967 (PMC7767894; doi:10.3389/fcell.2020.592967)

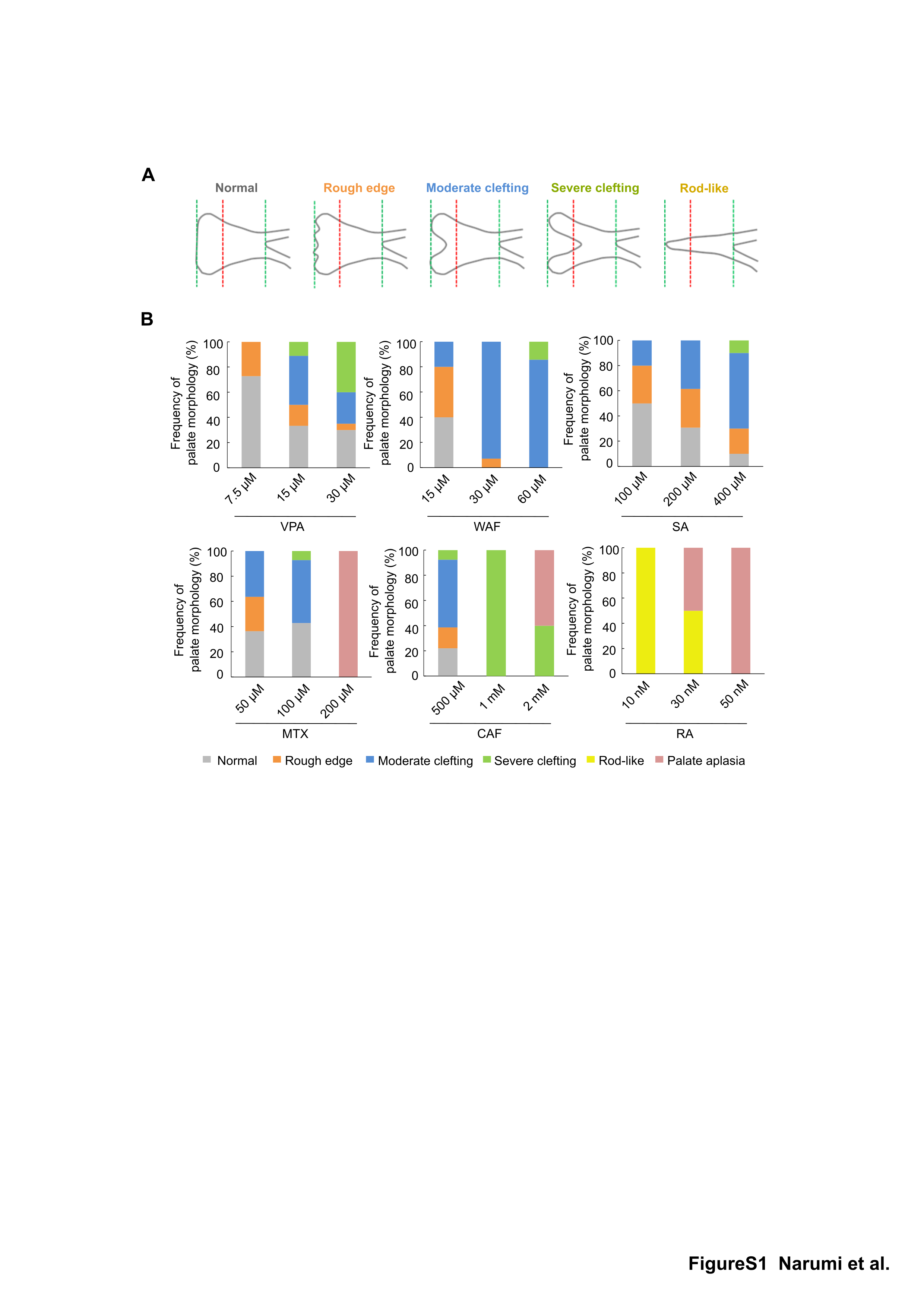

Supplement: Supplementary Figure 1 — Phenotypic severity of teratogen treatment in a dose-dependent manner. (A) Definition of phenotypic severity. Anterior is to the left. Green dotted lines indicate the anterior (left) and posterior (right) edge of the zebrafish palate, respectively. The palate length between the green dotted lines is defined as 100% length. Red dotted line indicates 40% length line of the palate length. Each phenotype was defined as follows: rough edge; several small nicks existing at the anterior edge, moderate clefting; under 40% clefting at the center of the palate, severe clefting; over 40% clefting of at the center of the palate, and rod-like; the palate showing rod-like structure. (B) Frequency of palatal defects in dose-dependent manner. n = 11 (VPA 7.5 μM), 18 (VPA 15 μM), 20 (VPA 30 μM), 10 (WAF 15 μM), 14 (WAF 30 μM), 14 (WAF 60 μM), 10 (SA 100 μM), 13 (SA 200 μM), 13 (SA 400 μM), 11 (MTX 50 μM), 14 (MTX 100 μM), 8 (MTX 200 μM), 13 (CAF 500 μM), 17 (CAF 1 mM), 10 (CAF 2 mM), 16 (RA 10 nM), 12 (RA 30 nM), 11 (RA 50 nM). [file Image_1.TIF]

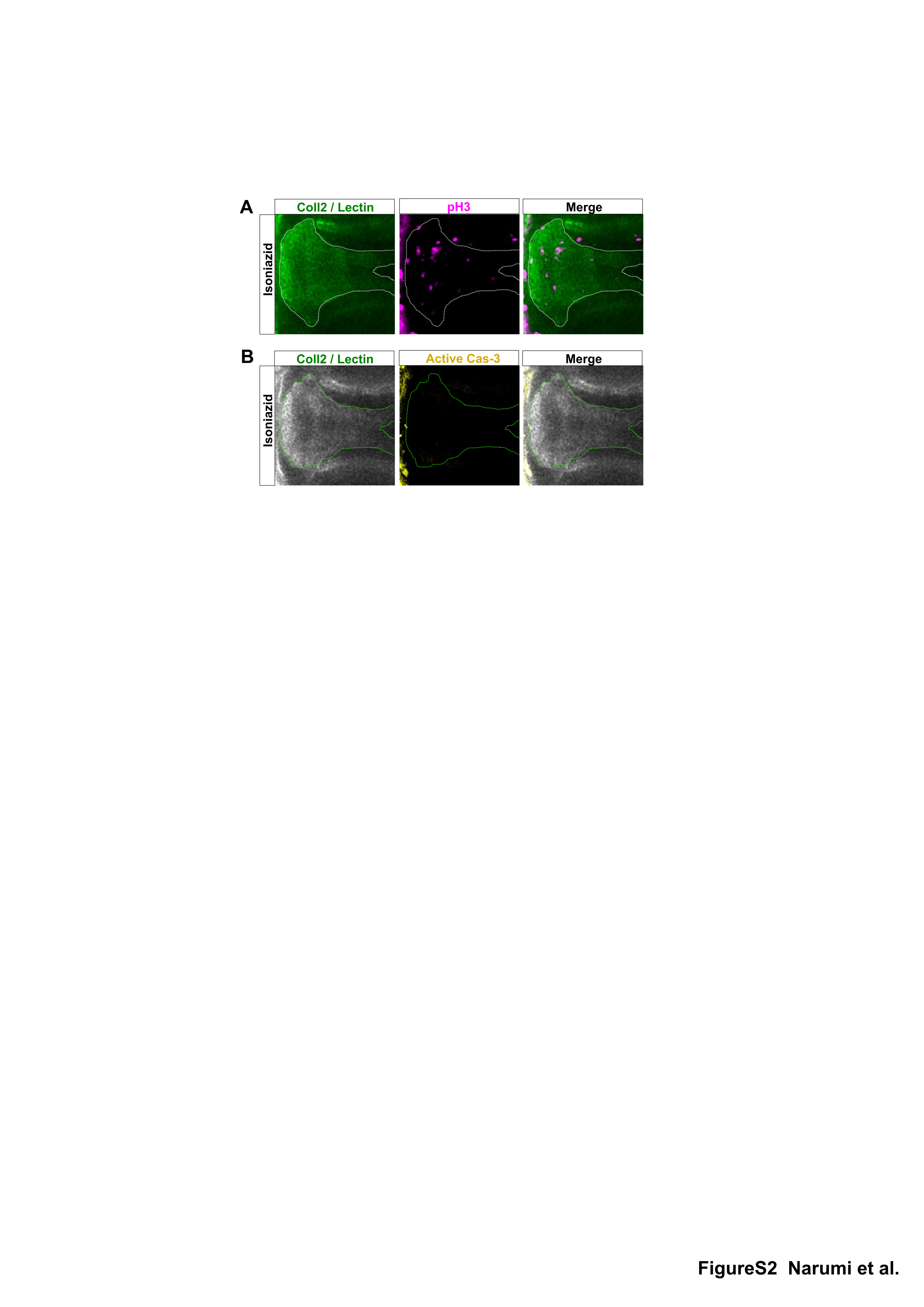

Supplement: Supplementary Figure 2 — Proliferation and apoptosis in the palate of INA-exposed embryos. (A) Immunofluorescence images of proliferative cells in the palate. Control is the same as Figure 3A. Embryos were treated with isoniazid (1 mM) and then examined by fluorescent immunohistochemistry. No striking difference was observed between the control and treated embryos. Green indicates cartilage cells double stained with anti-coll2 antibody and lectin PNA. White dotted lines trace the shape of the palate. Magenta indicates proliferative cells stained with anti-pH3 antibody. (B) Immunofluorescence images of apoptotic cells in the palate at 96 hpf. Control is the same as in Figure 3C. Embryos were treated with isoniazid (1 mM) and then examined by fluorescent immunohistochemistry. No striking difference was observed between the control and treated embryos. White indicates cartilage cells double stained with anti-coll2 antibody and lectin PNA. Green dotted lines trace the shape of the palate. Yellow indicates proliferative cells stained with anti-caspase3 antibody. Scale bar: 50 μm. [file Image_2.TIF]

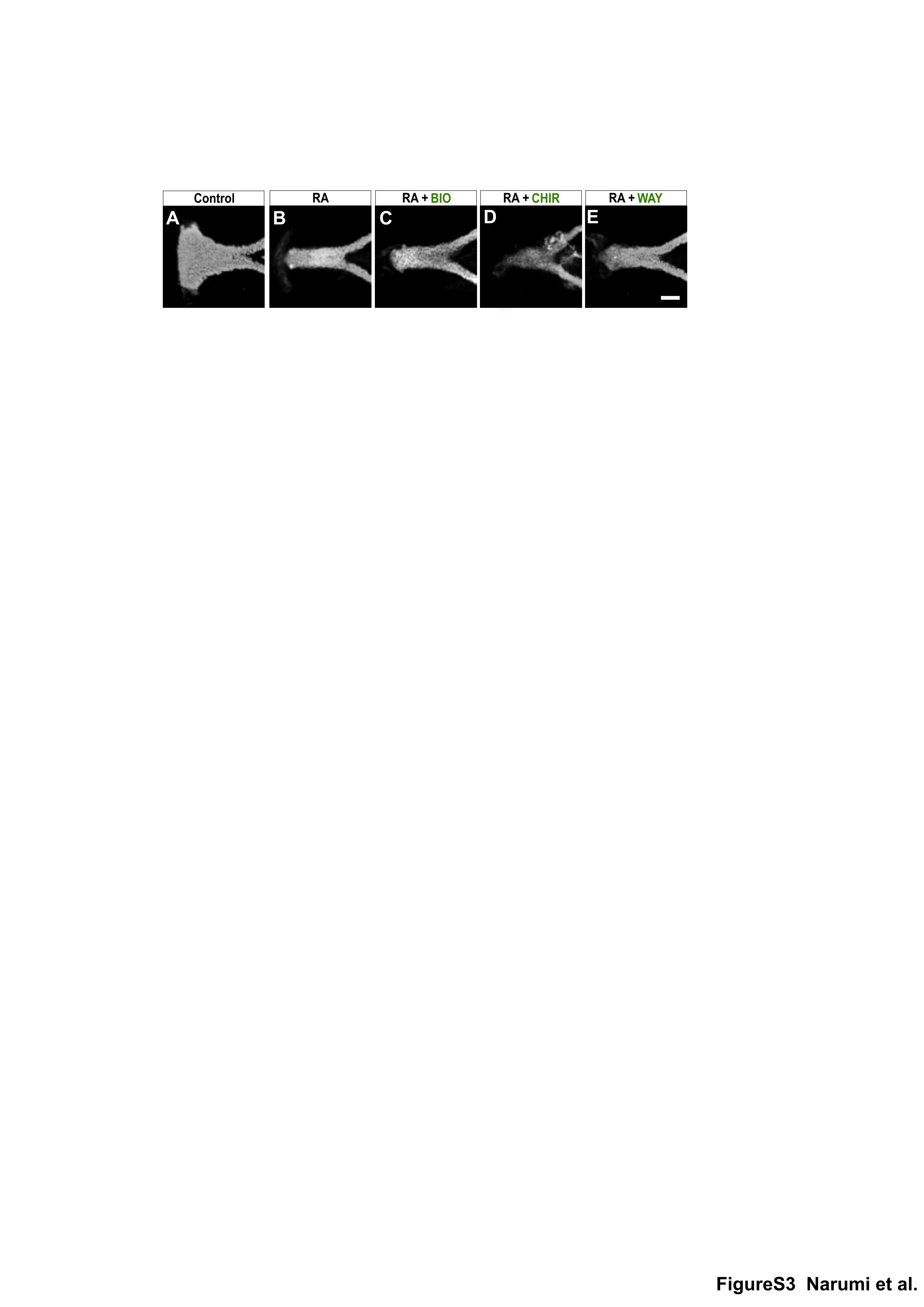

Supplement: Supplementary Figure 3 — Rod-like phenotype was not rescued by Wnt agonists. (A–E) Fluorescence images of palate at 96 hpf. Nuclei of cartilage cells were stained with DAPI. Embryos were treated with retinoic acid (RA, 10 nM). (A,B) Retinoic acid (RA) induced rod-like phenotype. (C–E) The rod-like phenotype was not rescued by combinatorial treatment with Wnt agonists [BIO (100 nM), CHIR99021 (300 nM) or WAY-262611 (250 nM)]. Scale bar: 50 μm. [file Image_3.TIF]
